# Amid Growing Evidence of Conflicts of Interest and Obdurate Groupthink in Medical Journals, Researchers Must Entertain Contrarian Ideas

**DOI:** 10.7759/cureus.77895

**Published:** 2025-01-23

**Authors:** Raphael Lataster, Peter Parry

**Affiliations:** 1 Faculty of Arts and Social Sciences, University of Sydney, Sydney, AUS; 2 Faculty of Medicine, University of Queensland, Brisbane, AUS

**Keywords:** contrarian ideas, groupthink, incorrect paradigms, taboo science, the science

## Abstract

Mainstream medicine, like other academic fields, is shaped by prevailing paradigms and the dominant narratives they create. Over the past half-century, these paradigms have increasingly reflected the growing commercial influence of the pharmaceutical industry. Dominant narratives are closely tied to groupthink, to which medical journals are often subject. In addition, more “prestigious” medical journals tend to have further financial conflicts of interest with the pharmaceutical industry. These dynamics limit scientific progress by suppressing awareness of the iatrogenic aspects of industry products and the benefits of alternative non-patentable and unpatentable medical products and therapeutic interventions. Journals need to adopt a more open policy to manuscripts that encompass contrarian perspectives to dominant narratives while still adhering to time-tested scientific values and methods.

## Editorial

Thomas Kuhn proposed that scientific endeavor is subject to “paradigms” that restrict what ideas are considered valid [1]. Thus, scientists and the peer-reviewed literature are as influenced by groupthink processes as by dispassionate rationality. There is the open-minded methodology of science, following data and evidence wherever it leads, even if surprising, contrarian, and disconcerting. Then there is “The Science,” which, in recent years, we were told to follow as established, settled, and having the imprimatur of authority.

Groupthink theories can be vociferously held and enforced with brutal censorship, as was the case with Semmelweis for daring to suggest doctors wash their hands [2]. During the past half-century, the profitability of large pharmaceutical companies has enabled them to dispense enormous financial investments into research, universities, medical education, medical journals, political parties, drug regulators, medical colleges and associations, and supranational institutions such as the World Health Organization. They can create the medical groupthink consensus.

Internal pharmaceutical industry documents released in litigation from criminal trials, where the industry has been fined $122 billion since 2000 [3,4], have revealed companies invest in shaping narratives to dominate a particular medical field in favor of their products, understating harms and overstating benefits [5]. Conflicts of interest now bedevil every level of pharmaceutical/medical science. Beginning with the obvious, pharmaceutical companies tend to oversee the trials for their own products [6]. Less obvious but more concerning, the large pharmaceutical companies provide the majority of funding to the regulators tasked with considering the evidence of clinical trials and granting or denying licensure [7].

The results of trials are disseminated to the wider scientific community via the major medical journals. A few journal chief editors have stated that their publications are effectively part of Big Pharma’s marketing departments [8-11]. Editors of major medical journals have received payments from large pharmaceutical companies [12]. Now it is discovered that peer reviewers have also received payments [13]. And as well-known now, pharmaceutical companies make payments to doctors [14], with one study noting that such “financial conflicts of interest may influence physician prescribing” and “remain pervasive” [15]. It is not surprising that pharmaceutical product recalls for adverse risk/benefit profiles are numerous, but take years to be actioned [16].

With these ubiquitous disclosed and undisclosed financial conflicts of interest, claims made by The Science’s gatekeepers about what is “acceptable” scientific thinking mandate a healthy skepticism, particularly when such claims are so quickly refuted. For instance, a recent exchange in the UK journal Public Health in Practice argued that it is wrong “to discredit scientists who hold opposing views.” The article also noted that “an abundant literature has since depicted a far more nuanced picture” regarding recently approved pharmaceutical products [17]. Furthermore, it emphasized the need for intellectual humility, acknowledging that “absolute certainty will almost certainly remain out of reach,” while also pointing out the prevalence of “reverse misinformation” - claims initially labeled as misinformation but later proven true [18]. A similar exchange took place in the Polish Annals of Medicine [19,20]. There is also debate about the benefits of low-carbohydrate diets. On the one hand, mainstream medical organizations state they are “unsafe and should be avoided,” with a lack of long-term safety data [21]. And yet, as published in The Lancet, the “ketogenic diet has been widely and successfully used to treat children with drug-resistant epilepsy since the 1920s” [22] and is showing efficacy for other psychiatric disorders [23]. As with most fields of knowledge, medical science is constantly evolving, and disagreement is both reasonable and rife.

A contrarian idea outside the norm can often be seen as crazy - much like the once-suppressed notion of washing hands before surgery, now an obvious and simple practice, yet dismissed in the past due to conflicts of interest [24]. Semmelweis’ theory of infectious “particles” preceded the paradigm shift to germ theory, and the medical establishment vehemently rejected his theory despite his results in reducing childbed fever deaths by orders of magnitude with chlorine solution handwashing by doctors under his supervision. Semmelweis developed severe depression subsequent to pervasive ridicule by his profession and was committed to a mental asylum, where he died after being beaten by his attendants. Considered a misinformation merchant in his own time, he is now an icon of medical science, innovation, and courage.

Contrarian ideas are necessary. Janis, the originator of the theory of “groupthink,” noted that “mindguards” are members of a group, like the establishment physicians and surgeons of Semmelweis’ era, who rigorously censor dissenters so as to uphold established orthodox theory and ideas [25].

A remedy to errors of groupthink is to uphold the right of dissenters to be heard [26]. Ideally, medical journals would actively encourage the discussion of contrarian ideas, perhaps with dedicated sections to such or by seeking responses from researchers with differing views. Unfortunately, groupthink in scientific peer review has been established as a bias factor [27], with the risk that editors and peer reviewers act as unscientific “mindguards.” “Censorship and silencing practices” in the scientific literature and online discourse, amounting to groupthink mindguarding activity, during the COVID era have been surveyed, noting “deleterious consequences” to clinicians and researchers who questioned the prevailing orthodoxy and consequent damage to the public’s trust in medical science [28]. Moreover, the recent US Congress House of Representatives’ Select Subcommittee on the Coronavirus Pandemic 520-page report fully or partly vindicated contrarian perspectives on many aspects of public health messaging during the pandemic [29].

Academic publishing is easier when sticking to approved narratives, but this retards progress. Contrarian ideas are vital to the expansion of knowledge. Epistemologists will understand that the search for truth is akin to carving a marvelous sculpture out of a block of marble. Much must be discarded, but that is part of the process. We chip away the detritus until we at last stumble upon the beauty that is the truth within.

Something is rotten in the Academy. We need to improve. One way to improve is to properly address financial and other conflicts of interest. Another way is to entertain contrarian ideas, to indulge those occupied with “taboo science,” while still adhering to time-tested scientific principles and methods.
